# MAVS ubiquitination by the E3 ligase TRIM25 and degradation by the proteasome is involved in type I interferon production after activation of the antiviral RIG-I-like receptors

**DOI:** 10.1186/1741-7007-10-44

**Published:** 2012-05-24

**Authors:** Céline Castanier, Naima Zemirli, Alain Portier, Dominique Garcin, Nicolas Bidère, Aimé Vazquez, Damien Arnoult

**Affiliations:** 1 INSERM UMR_S 1014, Hôpital Paul Brousse, Bâtiment Lavoisier, 14 avenue Paul Vaillant Couturier, 94807 Villejuif cedex, France; 2 Université Paris-Sud P11, France; 3 Department of Microbiology and Molecular Medicine, University of Geneva School of Medicine, 1 rue Michel-Servet, 1211 Geneva, Switzerland

**Keywords:** MAVS, RIG-I-like receptors, TRIM25, ubiquitination

## Abstract

**Background:**

During a viral infection, the intracellular RIG-I-like receptors (RLRs) sense viral RNA and signal through the mitochondrial antiviral signaling adaptor MAVS (also known as IPS-1, Cardif and VISA) whose activation triggers a rapid production of type I interferons (IFN) and of pro-inflammatory cytokines through the transcription factors IRF3/IRF7 and NF-κB, respectively. While MAVS is essential for this signaling and known to operate through the scaffold protein NEMO and the protein kinase TBK1 that phosphorylates IRF3, its mechanism of action and regulation remain unclear.

**Results:**

We report here that RLR activation triggers MAVS ubiquitination on lysine 7 and 10 by the E3 ubiquitin ligase TRIM25 and marks it for proteasomal degradation concomitantly with downstream signaling. Inhibition of this MAVS degradation with a proteasome inhibitor does not affect NF-κB signaling but it hampers IRF3 activation, and NEMO and TBK1, two essential mediators in type I IFN production, are retained at the mitochondria.

**Conclusions:**

These results suggest that MAVS functions as a recruitment platform that assembles a signaling complex involving NEMO and TBK1, and that the proteasome-mediated MAVS degradation is required to release the signaling complex into the cytosol, allowing IRF3 phosphorylation by TBK1.

## Background

Upon infection, viruses are rapidly recognized by the innate immune system through germ line-encoded pattern-recognition receptors (PRRs) [1]. Several classes of PRR, including Toll-like receptors (TLRs) and RIG-I-like receptors (RLRs), recognize viral components and directly activate immune cells. The RLRs are comprised of RIG-I and MDA-5 (melanoma differentiation-associated gene-5) that are cytosolic helicases sensing viral RNA [2]. Importantly, RIG-I and MDA-5 contain two CARDs (Caspase Activation and Recruitment Domains) [1,2]. The ATPase activity of both helicases as a result of binding to their ligands is required for conformational changes that lead to the exposure of the CARDs otherwise masked by the C-terminal regulatory domain. This conformational change is required for a putative interaction with the CARD domain of the mitochondrial adaptor MAVS (also known as IPS-1, Cardif or VISA) [3-6]. MAVS then activates two cytosolic protein kinase complexes, one consisting of the "noncanonical" IKK-related kinase TBK1 (TANK-binding kinase 1) or IKK-i/ε (inducible IκB kinase) associated with various adaptor proteins like TANK (TRAF family member associated NF-κB activator), NAP1 (NAK-associated protein 1) and NEMO (NF-κB Essential MOdulator), and the other comprising IKKα, IKKβ and NEMO [1]. The TBK1 complex leads to phosphorylation and dimerization of the transcription factors IRF3 and IRF7, which translocate to the nucleus and bind to IFN-stimulated response elements (ISREs), thereby resulting in the expression of type I IFN genes and a set of IFN-inducible genes. The IKK complex activates NF-κB, subsequently promoting the expression of pro-inflammatory cytokines [1].

Interestingly, it has been reported that MAVS must be localized to mitochondria to exert its function [5], suggesting that the mitochondrial environment is required for signal transduction following RLR activation. In agreement with this hypothesis, we recently reported that mitochondrial dynamics regulate MAVS-mediated signaling [7]. Nevertheless, the regulation of MAVS in the RLR pathway remains unclear.

Here, we report that RLR activation induces a selective proteasomal degradation of the larger MAVS isoform, subsequent to its ubiquitination on lysine 7 and 10 by the E3 ubiquitin ligase TRIM25. Surprisingly, this MAVS degradation seems to be required for the downstream signaling leading to type I IFN production, since its inhibition with a proteasome inhibitor prevents IRF3 activation. Importantly, we observed that prevention of the selective MAVS degradation leads to a retention at the mitochondria of NEMO and TBK1. Thus, our results suggest that MAVS acts as a recruitment platform for the assembly and activation of a signaling complex, and that MAVS degradation is likely required to release this signaling complex into the cytosol for IRF3 phosphorylation and ensuing type I IFN production.

## Results

### RLR activation promotes a selective degradation of the larger MAVS isoform concomitantly with downstream signaling

**Figure 1:**
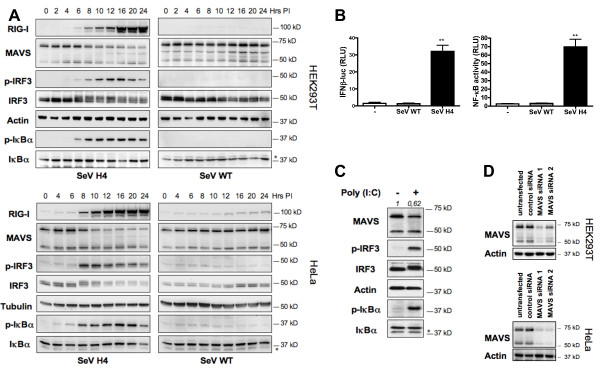
**The larger MAVS isoform is selectively degraded concomitantly with downstream signaling following RLR activation**. **(A) **HEK293T or HeLa cells were infected with SeV WT or H4, and at various times after infection RIG-I, MAVS, p-IRF3, IRF3, p-IκBα and IκBα were analyzed in cell extracts by immunoblotting. Actin was used as a protein loading control. **(B) **HEK293T cells were transfected with either an IFN-β promoter reporter or with NF-κB reporter as well as with renilla luciferase as an internal control. Twenty hours after transfection, cells were infected with SeV WT or SeV H4 or else left non-infected (-). Luciferase assay was performed 8 hr after infection and was normalized using renilla luciferase activity. Data represent means ± SD (*n *= 3). **(C) **HeLa cells were transfected with HMW Poly(I:C) (1 μg/ml) for 9 hr and then, cell extracts were analyzed by immunoblotting. * Probable non-specific protein bands. Values represent the ratio of the larger MAVS isoform band normalized with respect to the loading control, analyzed with ImageJ software. **(D) **Control or MAVS siRNAs were transfected into HEK293T or HeLa cells. Knockdown of MAVS was confirmed by immunoblotting 72 hr later.

To gain insight into the function and regulation of MAVS following RLR activation, we investigated the kinetics of signaling downstream of RIG-I by infecting HEK293T or HeLa cells with the Sendaï virus (SeV) H4 strain [8], a strain composed mostly of small, copy-back defective interfering genomes and whose infection overproduces short uncapped 5'-triphosphate RNAs that are specific ligands for RIG-I [2]. Accordingly, RIG-I has been reported to be essential for the production of type I IFN in response to SeV [9]. As a control, a wild-type (WT) SeV strain was used. Immunoblot analyses at different time points following infection of cells with these SeV strains confirmed that, unlike SeV WT, SeV H4 activates the RLR pathway as observed by the phosphorylation of both IRF3 and the NF-κB inhibitor IκBα (Figure 1A). RLR activation led to type I IFN production as assessed by the up-regulation of RIG-I in SeV H4-infected cells (Figure 1A). Next, in assays where a luciferase reporter was either under the control of the IFN-β promoter or driven by three copies of an NF-κB enhancer, SeV H4 activated not only the IFN-β promoter but also NF-κB, in contrast to SeV WT (Figure 1B).

In different cell lines, including HEK293T, HeLa (Figure 1A), A549, Huh7 or Jurkat cells (data not shown), MAVS is expressed as two major isoforms, as previously reported [5], and siRNAs raised against MAVS knock down the expression of the different isoforms (Figure 1D). Interestingly, following SeV H4 but not SeV WT infection, the larger isoform was degraded, whereas the shorter isoform was not affected (Figure 1A). Intriguingly, degradation of the larger MAVS isoform was concomitant with the phosphorylation of both IRF3 and IκBα, suggesting that this degradation could be connected to downstream signaling. Similar degradation was also observed in polyinosine-polycytidylic acid (poly(I:C))-transfected HeLa cells (Figure 1C) (in that case, poly(I:C) are sensed by MDA-5 [9]) or in Vesicular Stomatitis Virus (VSV)-infected cells (data not shown). Specific degradation of the larger MAVS isoform was also remarked after lysis in a buffer containing Sodium Dodecyl Sulfate (SDS) (Additional file 1) ruling out the hypothesis that the larger MAVS isoform moves into an insoluble fraction following RLR activation.

To assess whether this specific MAVS degradation is not a consequence of a feedback loop mediated by the production of type I IFN, cells were either treated with IFN-α2 or IFN-β, or treated after infection with a neutralizing antibody raised against IFNAR1, a chain of the IFN-α/β receptor. Unlike RLR activation, we observed that stimulation of cells with IFN did not promote MAVS degradation and inhibition of IFNAR1 did not prevent the degradation as well (Additional file 2) indicating that MAVS degradation is not a consequence of the production of type I IFN.

### MAVS degradation upon RLR activation is independent of a specific protease

The finding that the larger MAVS isoform is selectively degraded following RLR activation prompted us to investigate the mechanism of this degradation. First, by RT-PCR, we did not observe any differences in MAVS mRNAs in infected cells in comparison to non-infected cells (data not shown) suggesting a post-translational regulation of MAVS. It has been reported that MAVS can be processed and inactivated by a specific cleavage triggered by the hepatitis C virus serine protease NS3-4A or by cellular caspases activated by various pro-apoptotic signals [4,10]. A degradation of the larger MAVS isoform occurred following SeV H4, but not WT, infection (Figure 1A), implying that MAVS is not cleaved by a specific SeV protease. In addition, MAVS degradation occurred independently of caspases, since it was not prevented by the broad spectrum caspases inhibitor zVAD-fmk (Additional file 3A). By contrast, MAVS cleavage, as well as PARP processing, were abrogated in cells undergoing apoptosis in the presence of the caspase inhibitor (Additional file 3A). The caspase inhibitors zVAD-fmk and qVD-fmk had no effect on activation of IFNβ promoter or NF-κB as assessed in luciferase assays (Additional file 3B). Likewise, treatment with Leupeptin and Pepstatin, which are inhibitors of trypsin-like/some serine proteases and acid proteases, respectively, did not hamper MAVS degradation and downstream signaling (Additional files 3C, D).

### RLR activation triggers MAVS ubiquitination and degradation by the proteasome

**Figure 2:**
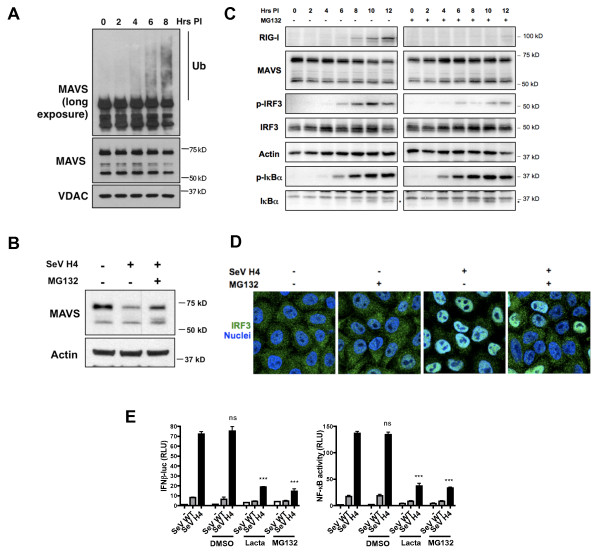
**RLR activation induces proteasomal degradation of the larger MAVS isoform after its polyubiquitination**. **(A) **HeLa cells were infected with SeV H4 in the presence of MG132. At different times after infection, MAVS and its ubiquitination were analyzed in mitochondrial extracts by immunoblotting with a short and a long exposure respectively. **(B) **HeLa cells were infected with SeV H4 in the presence or the absence of MG132. Nine hours after infection, MAVS was analyzed in cell extracts by immunoblotting. **(C) **HEK293T cells were infected with SeV H4 in the presence or the absence of MG132, then at various times after infection, RIG-I, MAVS, p-IRF3, IRF3, p-IκBα and IκBα were analyzed in cell extracts by immunoblotting. **(D) **HeLa cells were infected or not with SeV H4 for 8 hr in the presence or the absence of MG132. Next, nuclear translocation of IRF3 was assessed by immunofluorescence. **(E) **HEK293T cells were transfected with either an IFN-β promoter reporter or with NF-κB reporter as well as with renilla luciferase as an internal control. 24 hr after transfection, cells were infected with SeV WT or SeV H4 or else left non-infected (-) and treated with different proteasome inhibitors. Luciferase assay was performed 8 hr after infection and was normalized using renilla luciferase activity. Data represent means ± SD (*n *= 3).

Since protease inhibitors had no effect on MAVS degradation, we hypothesized that MAVS might be degraded by the proteasome. It is believed that polyubiquitin chains linked through lysine at position 48 of ubiquitin (Lys 48) target protein substrates for degradation by the proteasome, whereas polyubiquitin chains of alternative linkages (such as Lys 63) carry out signaling functions independent of proteolysis [11]. Analysis of MAVS in the mitochondrial fraction from SeV H4-infected cells demonstrated that MAVS is rapidly ubiquitinated during infection (Figure 2A), as previously described [12]. Importantly, treatment with the proteasome inhibitor MG132 prevented degradation of the larger MAVS isoform (Figure 2B), signifying that following RLR activation, the larger MAVS isoform is ubiquitinated then selectively degraded by the proteasome. Surprisingly, the proteasome inhibition not only impaired MAVS degradation following RLR activation but also prevented IRF3 phosphorylation (Figure 2C), its nuclear translocation (Figure 2D) and ensuing type I IFN production, as assessed by the lack of RIG-I expression (Figure 2C) or by luciferase assays (Figure 2E). Interestingly, proteasome inhibition did not impair IκBα phosphorylation (Figure 2C), indicating that prevention of MAVS degradation has no impact on IKK activation. Nevertheless, as expected, MG132 treatment inhibited NF-κB activation because IκBα is degraded by the proteasome once phosphorylated (Figures 2C, E) [11]. Finally, the use of lactacystin, another proteasome inhibitor, also prevented activation of the IFN-β promoter (Figure 2E). Together, our observations strongly suggest that the proteasome-mediated degradation of MAVS is required for the signal transduction that leads to IRF3 activation and ensuing type I IFN production.

### The E3 ubiquitin ligase TRIM25 targets MAVS for ubiquitination and degradation

**Figure 3:**
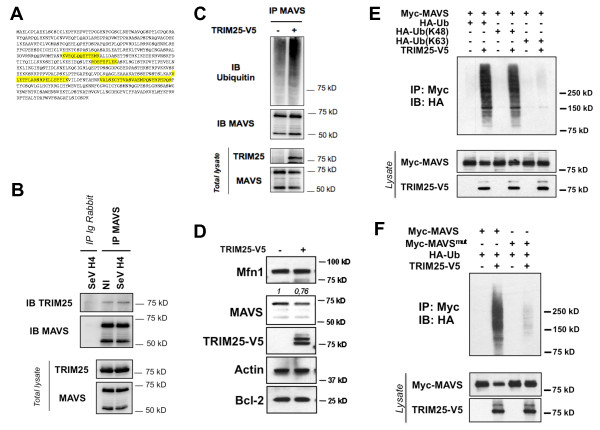
**The E3 ubiquitin ligase TRIM25 catalyzes Lys 48-linked ubiquitination of MAVS**. **(A) **The sequence of TRIM25 and the matching endogenous peptides (highlighted in yellow) were identified by mass spectrometry. **(B) **HEK293T cells were infected with SeV H4 in the presence of MG132 for 6 hr. Next, endogenous MAVS was immunoprecipitated from cell extracts; the presence of MAVS and TRIM25 was examined by immunoblotting. **(C) **HEK293T cells were transfected with TRIM25-V5 or control plasmid for 24 hr. Next, endogenous MAVS was immunoprecipitated in denaturing conditions from cell extracts with specific antibody; the presence of MAVS and its ubiquitination was examined by immunoblotting. **(D) **HEK293T cells were transfected with TRIM25-V5 or control plasmid, and 72 hr after transfection, Mfn1, MAVS, V5 (TRIM25) and Bcl-2 were analyzed in cell extracts by immunoblotting. Values represent the ratio of the larger MAVS isoform band normalized with respect to the loading control. **(E) **HEK293T cells were transfected with the indicated plasmids. Twenty-four hours after transfection, immunoprecipitation and immunoblot analysis were performed with the indicated antibodies (*upper panel*). Expression of the proteins was examined by immunoblots with the indicated antibodies (*lower panel*). **(F) **Experiment was performed as in E. Myc-MAVS^mut^: Myc-MAVS^(K7R/K10R) ^mutant.

To identify an E3 ubiquitin ligase involved in the selective degradation of MAVS, cells were infected with SeV H4 for four hours, and the purified MAVS complex after immunoprecipitation was analyzed by mass spectrometry. Four E3 ubiquitin ligases were identified by mass spectrometry, and among them TRIM25 drew our attention (Figure 3A) because this E3 ubiquitin ligase was already known to be involved in the RLR pathway. Indeed, TRIM25 induces the Lys 63-linked ubiquitination of RIG-I to stabilize the interaction with MAVS [13] but TRIM25 is also capable of promoting Lys 48-linked ubiquitination and degradation of proteins [14,15]. Interaction between MAVS and TRIM25 was confirmed by co-immunoprecipitation of endogenous MAVS, and the interaction was slightly enhanced after SeV H4 infection (Figure 3B). Transfection of TRIM25 increased MAVS ubiquitination (Figure 3C) and promoted specifically a modest but significant degradation of the larger MAVS isoform (Figure 3D). In sharp contrast, two other mitochondrial proteins anchored into the outer membrane, namely Mfn1 and Bcl-2, remained unaffected (Figure 3D). Importantly, TRIM25 catalyzed MAVS ubiquitination with WT ubiquitin and ubiquitin-K48, but not with ubiquitin-K63 (Figure 3E). Because only the larger MAVS isoform is degraded following RLR activation, we hypothesized that the lysine residues that are targeted by TRIM25 to promote MAVS ubiquitination are only present in the larger isoform. The shorter MAVS isoform is a truncated form which lacks the N-terminus but retains the C-terminal transmembrane domain (unpublished observation and [16]). Sequence analysis revealed that two lysines (K7 and K10) are present in the larger isoform only. We, therefore, mutated K7 and K10 and investigated whether the mutation affects TRIM25-mediated MAVS ubiquitination. While a single mutation (K7R or K10R) partially reduces the ubiquitination profile of MAVS (data not shown), MAVS ubiquitination was strongly inhibited when a double mutation was realized (K7R/K10R) (Figure 3F). Accordingly, MAVS degradation was also prevented (Figure 3F). Because of K7's and K10's close proximity, we speculate that they likely compensate for each other. Together, these data suggest that TRIM25 targets MAVS at K7 and K10 for ubiquitination and degradation.

**Figure 4:**
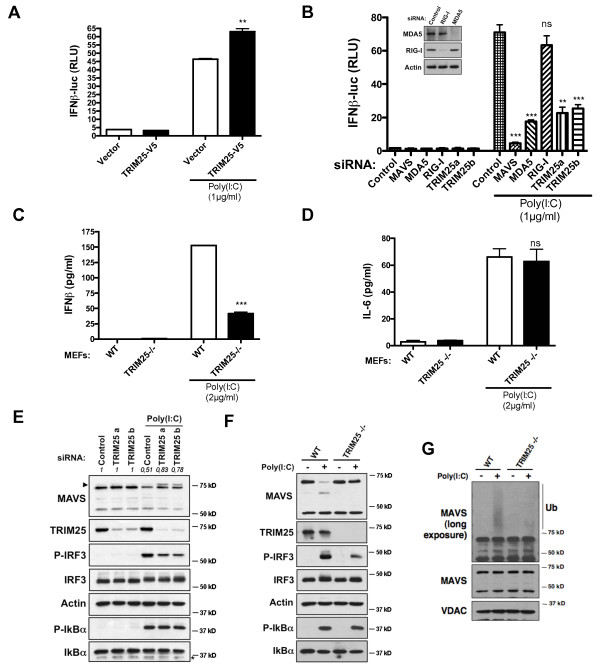
**Involvement of TRIM25 in the regulation of MAVS**. **(A) **HeLa cells were transfected with empty or TRIM25-V5 vector and co-transfected with an IFN-β promoter reporter as well as with renilla luciferase as an internal control. Twenty-four hours later, cells were transfected or not with HMW Poly(I:C) (1 μg/ml). Luciferase assay was performed 8 hr after transfection and was normalized using renilla luciferase activity. Data represent means ± SD (*n *= 3). **(B) **HeLa cells were transfected with control, MAVS, RIG-I and TRIM25 siRNAs for 48 hr, next transfected with an IFN-β promoter reporter as well as with renilla luciferase as an internal control. Twenty-four hours later, cells were transfected or not with Poly(I:C) (1 μg/ml). Luciferase assays were performed 8 hr after transfection and normalized using renilla luciferase activity. Data represent means ± SD (*n *= 3). The knockdown efficiency of MDA-5 and RIG-I was evaluated by immunoblot. For the knockdown of MAVS and TRIM25, see Figures 1D and 4E respectively. **(C) **Concentrations of mouse IFN-β in cellular supernatant from WT or *TRIM25*^-/- ^MEFs, 9 hr after transfection with 2 μg/ml of Poly(I: C). IFN-β concentrations were assessed by ELISA. Data represent means ± SD (*n *= 2). **(D) **Concentrations of mouse IL-6 in cellular supernatant from WT or *TRIM25*^-/- ^MEFs, 9 hr after transfection with 2 μg/ml of Poly(I: C). IL-6 concentrations were assessed by ELISA. Data represent means ± SD (*n *= 2). **(E) **HeLa cells were transfected with control or TRIM25 siRNAs for 72 hr. Then, cells were transfected or not with Poly(I:C) (1 μg/ml), and 9 hr after transfection TRIM25, MAVS, p-IRF3, IRF3, p-IκBα and IκBα were analyzed in cell extracts by immunoblotting. Actin was used as a protein loading control. Arrow indicates the phosphorylated state of MAVS. Values represent the ratio of the larger MAVS isoform band normalized with respect to the loading control. **(F) **WT or *TRIM25*^-/- ^MEFs were transfected or not with Poly(I:C) (2 μg/ml), and 10 hr after transfection TRIM25, MAVS, p-IRF3, IRF3, p-IκBα and IκBα were analyzed in cell extracts by immunoblotting. Actin was used as a protein loading control. **(F) **WT or *TRIM25*^-/- ^MEFs were transfected or not with Poly(I:C) (2 μg/ml) in the presence of MG132. Three hours later, MAVS and its ubiquitination were analyzed in mitochondrial extracts by immunoblotting with a short and a long exposure respectively. VDAC was used as a protein loading control.

TRIM25 was described to act upstream of MAVS through the ubiquitination of RIG-I but not of MDA-5 [13]. To circumvent this trouble, we transfected HMW poly(I:C) into cells because this synthetic dsRNA analog is sensed by MDA-5 but not by RIG-I [9] (Figure 4B) and, thus, we noticed that transfection of TRIM25 augments activation of the IFNβ promoter once cells are stimulated (Figure 4A). In TRIM25 siRNA-transfected cells or in *TRIM25 -/- *MEFs, we observed that IFNβ production was significantly impeded after activation with poly(I:C), indicating that TRIM25 also regulates the RLR pathway independently of RIG-I (Figures 4B, C). Importantly, in *TRIM25 -/- *MEFs, IL-6 production was similar as in WT MEFs following transfection with poly(I:C) (Figure 4D), suggesting that TRIM25-mediated degradative ubiquitination of MAVS does not regulate NF-κB-induced cytokine production but only type I IFN synthesis (Figure 4C). Confirming this, immunoblot analyses showed that the knock down of TRIM25 inhibits the degradation of the larger MAVS isoform as well as the ensuing phosphorylation of IRF3, but not of IκBα after activation (Figures 4E, F). Furthermore, MAVS ubiquitination was strongly inhibited in *TRIM25 -/- *MEFs in comparison to WT MEFs after transfection with poly(I:C) (Figure 4G). Collectively, our data demonstrate that TRIM25 binds to MAVS and promotes its K48-linked ubiquitination and proteasome-mediated degradation to allow IRF3 but not NF-κB activation once RLRs are stimulated. Interestingly, the knock down of TRIM25 led to the appearance of a protein band above the larger MAVS isoform after stimulation in human cells (Figure 4E), and our results indicate that it corresponds to a phosphorylated state (Additional file 4). Appearance of this form of MAVS was a consequence of RLR activation (Additional file 4A) and treatment with λ phosphatase promoted its disappearance (Additional file 4B), confirming a phosphorylation of the larger MAVS isoform once stimulated by RLRs. Therefore, this observation suggests that to be degraded, the higher MAVS isoform is not only ubiquitinated but is also phosphorylated, likely explaining why the overexpression of TRIM25 only promotes limited degradation of endogenous MAVS (Figure 3D).

### IRF3 phosphorylation depends on a translocation of a signaling complex from mitochondria to cytosol upon proteasomal degradation of MAVS

**Figure 5:**
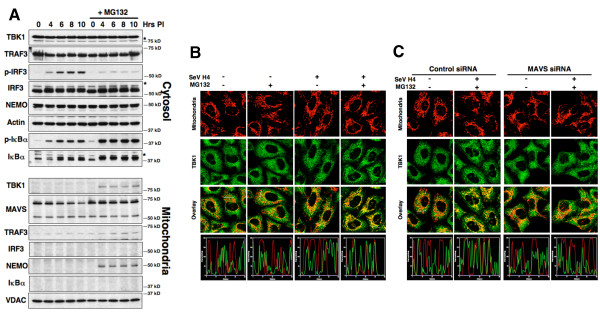
**MAVS degradation is required to release into the cytosol a signaling complex involved in IRF3 activation**. **(A) **HeLa cells were infected with SeV H4 in the presence or the absence of MG132. At various times after infection, cytosolic fraction and mitochondrial fraction were prepared. MAVS, TRAF3, p-IRF3, IRF3, p-IκBα, IκBα NEMO and TBK1 were analyzed in each fraction by immunoblotting. Actin and VDAC were used as a protein loading control for cytosol fraction and mitochondrial fraction, respectively. **(B) **HeLa cells were infected or not with SeV H4 for 8 hr in the presence or the absence of MG132. Co-localization (yellow) of TBK1 (green) with mitochondria (red) was observed by immunofluorescence. Line scans show the fluorescence intensities of TBK1 (green) and mitochondria (red) along the selected line. **(C) **Control or MAVS siRNA were transfected into HeLa cells for 72 hr. Then, HeLa cells were infected or not with SeV H4 for 8 hr in the presence or the absence of MG132. Co-localization (yellow) of TBK1 (green) with mitochondria (red) was observed by immunofluorescence. Line scans show the fluorescence intensities of TBK1 (green) and mitochondria (red) along the selected line.

**Figure 6:**
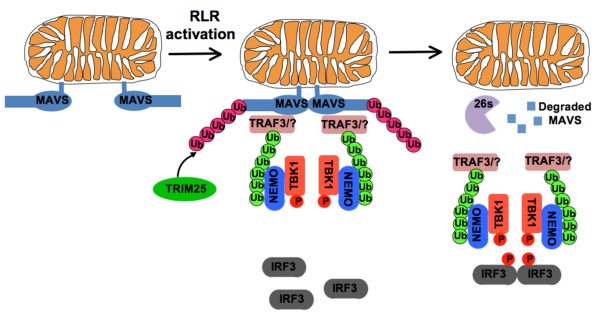
**IRF3 signaling depends on a translocation of signaling upon proteasomal-degradation of MAVS**. In non-activated cells, MAVS is associated to mitochondria through its C-terminal transmembrane domain. RLR activation induces MAVS oligomerization and aggregation [16] (to simplify the model, only two MAVS molecules are shown as forming an aggregate), then MAVS recruits TRAF3 and other E3 ubiquitin ligases which function to catalyze Lys 63-linked polyubiquitination of target proteins including TRAF3 itself. The Lys 63-linked polyubiquitin chains (in green) recruit NEMO which in turn binds to TBK1 and TBK1 is activated. Concomitantly, TRIM25 induces Lys 48-linked polyubiquitination (in red) of MAVS. The proteasomal degradation of MAVS results in the translocation of the MAVS-assembled complex into the cytosol where activated TBK1 phosphorylates IRF3 to promote type I IFN production.

Since our data indicate that following RLR activation the proteasome-mediated degradation of MAVS is required for IRF3 activation, we explored how this degradation is involved. Several proteins, such as TRAF3, NEMO and TBK1, have been reported to be critical effectors downstream of MAVS to trigger IRF3 phosphorylation and type I IFN production [1,11,17,18]. So we decided to investigate whether the prevention of MAVS degradation perturbs their localization within cells. Hence, cells were infected with SeV H4 in the presence or the absence of a proteasome inhibitor, and at different time points the presence of TRAF3, NEMO and TBK1 was studied in cytosolic and mitochondrial fractions. TRAF3, NEMO and TBK1 are cytosolic proteins, and their respective amounts remained unaffected by the infection in the presence or the absence of MG132 (Figure 5A). Like IRF3, TBK1 and NEMO were not found associated with purified mitochondria during infection, but interestingly, when MAVS degradation was inhibited, some TBK1 and NEMO were detected in the mitochondrial fraction (Figure 5A). Since both proteins are cytosolic, prevention of MAVS degradation likely promotes their retention into the mitochondrial fraction. This was confirmed by immunofluorescence studies. Indeed, in control or SeV H4-infected cells, TBK1 was diffusely localized in the cytosol, but when cells were pre-treated with MG132, a significant redistribution of TBK1 to mitochondria was observed in infected cells (Figure 5B). This relocation was dependent of MAVS, since MAVS knock down (Figure 1D) abrogated the association of TBK1 with mitochondria after infection (Figure 5C) and after cell fractionation, retention of both TBK1 and NEMO in the mitochondrial fraction was not observed (data not shown). A defect in the degradation of the larger MAVS isoform leads to a retention of both TBK1 and NEMO within the mitochondrial fraction and is subsequently linked to an inhibition of IRF3 phosphorylation (Figure 5A). We, therefore, propose that following RLR activation, MAVS allows the formation of a signaling complex composed at least of NEMO and TBK1, but this complex must be released into the cytosol consequently to MAVS degradation for TBK1 to phosphorylate IRF3 (Figure 6). Interaction between MAVS and the signaling complex is likely indirect because, as previously reported [19], we did not detect any association between MAVS and TBK1 or NEMO in co-immunoprecipitation experiments (unpublished observations). The signaling complex may also contain the E3 ubiquitin ligase TRAF3 because proteasome inhibition slightly increased the proportion of TRAF3 associated with purified mitochondria once RLRs are activated (Figure 5A). Moreover, while prevention of MAVS degradation impaired IRF3 phosphorylation following RLR activation (Figure 5A), it did not preclude IκBα phosphorylation (Figure 5A) suggesting again that MAVS degradation is not required for NF-κB signaling.

## Discussion

Previous studies have shown that the mitochondrial adaptor protein MAVS is essential to the RLR antiviral innate immune response [1,3-5,20,21]. Nevertheless, the function of MAVS, as well as its regulation in the RLR signaling pathway, has remained unclear. Very recently, it has been described that MAVS forms functional prion-like aggregates after viral infection and that these aggregates are required for the activation of IRF3 in the cytoplasm [16].

MAVS is expressed as two major isoforms, and here we demonstrate that RLR activation induces the specific proteasome-mediated degradation of the larger MAVS isoform after its polyubiquination, but, importantly, this degradation seems to be required for the downstream signaling that leads to type I IFN but not pro-inflammatory cytokines production. We identified the RING-finger E3 ubiquitin ligase TRIM25 as an E3 ubiquitin ligase that catalyzes Lys 48-linked ubiquitination of MAVS, leading to its degradation by the proteasome. TRIM25 has already been shown to play a critical role in the RLR pathway because it promotes Lys 63-linked ubiquitination of the CARD domain of RIG-I, but not of the related helicase MDA-5, to enhance and stabilize the interaction with the CARD domain of MAVS [13]. Nevertheless, it was reported that TRIM25 (also called Efp) targets the proteasome-mediated degradation of 14-3-3 σ and KLF5 [14,15] confirming that TRIM25 is capable of triggering Lys 48-linked ubiquitination as well. Hence, TRIM25 appears as an E3 ubiquitin ligase with a crucial dual role in the positive regulation of the RLR pathway through its function on RIG-I to favor the RIG-I/MAVS interaction as well as on MAVS to promote its proteasome-mediated degradation, which is likely required for IRF3 but not NF-κB activation. How MAVS is extracted from the mitochondrial outer membrane before proteasome degradation is currently unknown but it may involve the AAA ATPase cdc48/p97/VCP, thought to extract integral membrane proteins from the lipid bilayer and chaperone them to the proteasome, as it is the case for Mcl-1 and Mitofusins [22].

When TRIM25 was knocked down, degradation of the larger MAVS isoform that is observed following RLR activation was prevented, but, surprisingly, an accumulation of a phosphorylated even larger isoform of MAVS was detected as well. So it seems that to promote its degradation by the proteasome, MAVS must undergo a phosphorylation in addition to an ubiquitination, as is the case for the NF-κB inhibitor IκBα [11]. The nature and function of this phosphorylation, as well as the kinase(s) involved in this process, deserve further investigations.

Once the specific degradation of the larger MAVS isoform was prevented with a proteasome inhibitor, a retention of NEMO and TBK1, two essential mediators in type I IFN production, was observed in the mitochondrial fraction. While this mitochondrial retention of both proteins requires the presence of MAVS, we did not detect any direct interactions between MAVS and either NEMO or TBK1 (unpublished data), indicating an indirect association probably through ubiquitin chains. Indeed, a study has unveiled a key role of ubiquitin chains in IRF3 activation downstream of MAVS with NEMO functioning as a sensor of Lys 63 polyubiquitin chains to activate TBK1 [19]. In this model, an important question that remains to be resolved is the identity of the E3 ubiquitin ligase(s) that synthetizes the Lys 63 ubiquitin chains to mediate IRF3 activation by MAVS. A candidate is TRAF3, because this E3 ubiquitin ligase has been shown to be important for type I IFN production by RLRs [18,23], but in our hands the knock down of TRAF3 did not impair the activation of the IFNβ promoter (data not shown), suggesting that other E3 ubiquitin ligases can compensate the loss of TRAF3, as previously proposed [19]. Nevertheless, we found that a small fraction of TRAF3 is associated with mitochondria and this fraction was slightly increased when MAVS degradation was inhibited, indicating that TRAF3 may be a component of the signaling complex downstream of MAVS and composed of at least NEMO and TBK1.

## Conclusions

At the mitochondrial surface, MAVS likely serves as a recruitment platform for the assembly and activation of a signaling complex involving NEMO and TBK1 and required for IRF3 activation. Our observation that IRF3 was not phosphorylated and activated by TBK1 unless the NEMO/TBK1 complex translocates from the mitochondria into the cytosol after the proteasomal degradation of MAVS suggests that the cytoplasmic translocation of the MAVS-assembled signaling complex is required for an optimal IRF3 activation (Figure 6).

## Methods

### Cell culture and viral infection

HEK293T cells, HeLa cells and MEFs were cultured in standard conditions. *TRIM25-/- *MEFs were kindly provided by Dr. J.U. Jung (Department of Molecular Microbiology and Immunology, University of Southern California, Los Angeles, CA, USA). Sendaï virus (SeV) H4 and WT strains as well as infection protocol were described previously [7,8], and the Multiplicity of infection (MOI) was 40.

### Reagents

Proteasome inhibitors: MG132 (Calbiochem, Merck Chemicals Ltd. Nottingham, UK)) and Lactacystin (Calbiochem) were used at 10 μM and 25 μM, respectively. Proteases inhibitors: z-VAD-fmk (Calbiochem), qVD-fmk (Calbiochem), Leupeptin Hemisulfate (mpbio, Santa Ana, CA, USA) and Pepstatin A (Sigma-Aldrich, St. Louis, MO, USA) were used at 50 μM. Staurosporine (Sigma) was used at 2 μM. Interferons α and β (R&D Systems, Minneapolis, MN, USA) were used at 3,000 U/ml and 3,200 U/ml, respectively. HMW Poly(I:C) (Invivogen, San Diego, CA, USA) was transfected at 1 or 2 μg/ml. Lambda protein phosphatase (λ-PPase) was provided from New England Biolabs (NEB, Ipswich, MA, USA) (P0753S). Neutralizing anti-IFNAR1 (a gift of Dr. P. Eid) was used at 50 μg/ml.

### Protein extraction and immunoblot analysis

Cells were lysed in buffer-A (20 mM Tris-HCl (pH 7.4), 137 mM NaCl, 2 mM EDTA, 1% Triton X-100, 2 mM sodium pyrophosphate, 10% Glycerol, 25 mM β-glycerophosphate, 1 mM sodium orthovanadate) supplemented with the protease inhibitor mixture Complete (Roche Molecular Biochemicals, Meylan, France)). After incubation on ice for 20 minutes, a soluble extract was collected after centrifugation at 11,000 g for 10 minutes at 4°C. The lysate (20 μg) was boiled in SDS sample buffer and resolved by SDS-polyacrilamide gel electrophoresis. Immunoblot analysis was performed with specific antibodies and the Ag-Ab complexes were visualized by chemiluminescence (Immobilon Western, Merck Millipore, Billerica, MA, USA). For total cell extracts, cell were lysed in buffer-A supplemented with 3% SDS.

### Antibodies

The primary antibodies used in immunoblotting were as follows: mouse monoclonal anti-RIG-I (Alexis Biochemicals, San Diego, CA, USA, clone Alme-1{) (1:2,000 dilution), mouse monoclonal anti-Cardif/MAVS (Alexis Biochemicals, clone Adri-1) (1:4,000), rabbit polyclonal anti-rodent MAVS (Cell Signaling Technology, Danvers, MA, USA) (1:4,000), mouse monoclonal anti-actin (Sigma-Aldrich, St. Louis, MO, USA, clone AC-40) (1:5,000), rabbit polyclonal anti-MDA-5 (Alexis Biochemicals) (1:2,000), rabbit monoclonal anti-phospho-IRF3 (Cell Signaling Technology, clone 4D4G) (1:1,000), rabbit polyclonal anti-IRF3 (Santa Cruz Biotechnology, Santa Cruz, CA, USA) (1:1,000), rabbit monoclonal anti-IRF3 (Cell Signaling Technology) (1:2,000), mouse monoclonal anti-phospho-IκBα (Cell Signaling Technology, clone 5A5) (1:2,000), rabbit polyclonal anti-IκBα (Santa Cruz Biotechnology, C-21) (1:2,000), mouse monoclonal anti-IKKι/IKKε/TBK1 (Imgenex, San Diego, CA, USA, clone 72B587) (1:1,000), mouse monoclonal anti-PARP (BD, Franklin Lakes, NJ, USA, clone C2-10) (1:4,000), rabbit polyclonal anti-Stat1 (Upstate Biotechnology, Merck Millipore, Billerica, MA, USA) (1:1,000), rabbit polyclonal anti-phospho-Stat1 (Cell Signaling Technology, clone Tyr701) (1:1,000), rabbit polyclonal anti-TRAF3 (Santa Cruz Biotechnology, H-122) (1:500), mouse monoclonal anti-VDAC (Calbiochem, clone 89-173/025) (1:4,000), rabbit polyclonal anti-V5 (Sigma-Aldrich) (1:5,000), mouse monoclonal anti-HA (Sigma-Aldrich) (1:5,000). The antibody used in immunoprecipitation of endogenous MAVS was rabbit polyclonal anti-Cardif/MAVS (Alexis Biochemicals, clone AT107) and rabbit polyclonal anti-Myc (Sigma-Aldrich) for the immunoprecipitation of Myc-MAVS. The primary antibodies used for immunofluorescence microscopy were rabbit polyclonal anti-IRF3 (Santa Cruz Biotechnology) (1:500), polyclonal anti-TOM20 (Santa Cruz Biotechnology) (1:800) and mouse monoclonal anti-TBK1 (ProSci Incorporated, Poway, CA, USA, Clone 108A429) (1:400).

### Transfections and plasmids

Transfection of HEK293T cells was performed using the calcium phosphate precipitation method. Transfection of HeLa cells by DNA and poly(I:C) was performed using Lipofectamine 2000 (Invitrogen, Life Technologies, Grand Island, NY, USA **{**) and Oligofectamine (Invitrogen) was used for transfecting siRNAs. The plasmid for the expression of TRIM25-V5 was provided by Dr. J.U. Jung.

### Luciferase assays

Cells were plated in 24-well plates. On the second day, cells were co-transfected with 50 ng of firefly luciferase constructs under the control of the IFN-β promoter or driven by three copies of an NF-κB enhancer, and 10 ng of the renilla luciferase pRL-TK plasmid (Promega). The next day, cells were either infected by SeV or transfected with poly(I:C) for a few hours. Transfected cells were collected and luciferase activity was assessed using the Dual-luciferase reporter assay (Promega) on a Fluorostar Optima (BMG Labtech, Ortenberg, Germany). Each experiment was carried out in triplicates. For each sample, to obtain relative fluorescence units (RLU), firefly luciferase fluorescence units were normalized to renilla luciferase fluorescence units.

### Immunoprecipitation

Cell lysates were prepared in lysis buffer-B (50 mM Tris-HCl (pH 7.5), 140 mM NaCl, 5 mM EDTA, 5% glycerol, 1% Triton X-100, and 1% Nonidet P-40) supplemented with the protease inhibitor mixture Complete, on ice for 20 minutes. Soluble proteins (500 μg) were subjected to immunoprecipitation with an anti-MAVS (2.5 μg/ml), or rabbit anti-IgG antibody as a control or an anti-Myc antibody. An aliquot of the total lysates was included as a control. After one hour, 20 μl of equilibrated protein G-magnetic beads (Ademtech SA, Pessac, France) was added. Immunoprecipitation was carried out for one hour. The beads were then washed three times with buffer-B. Immune complexes were resolved by SDS-PAGE and immunoblotted.

### Lambda phosphatase test

Following immunoprecipitation, the G-magnetic beads were washed twice with the lysis buffer-B, and then twice with the lysis buffer B without EDTA and without the protease inhibitor mixture. Then, each sample was incubated with the reaction mixture (2.5 μl of reaction buffer provided with the λ Phosphatase kit (NEB), 2.5 μl MnCl2 (provided with the kit), 10 μl lysis buffer-B without EDTA/inhibitors and 10 μl λ-Phosphatase (NEB) or lysis buffer-B only for control) for 30 minutes at 30°C. Finally, Immunoblot and phosphorylation of MAVS were resolved by SDS-PAGE.

### Immunofluorescence microscopy

Cells grown in LabTek (Fisher Scientific, Illkirch, France) chambers were fixed for 10 minutes in 4% paraformaldehyde, followed by permeabilization with 0.15% Triton X-100 in PBS for 15 minutes. The cells were then incubated for one hour in blocking buffer (2% BSA in PBS) followed by incubation overnight with primary antibodies. Next, cells were washed three times for 10 minutes each in PBS, then incubated for 1 hr with Alexa Fluor secondary antibodies. Images were acquired using a Leica SP6 confocal microscope (Leica Microsystems, Wetzlar, Germany) through a 63x oil fluorescence objective.

Signal intensities from each channel were reconstructed by plotting pixel values of each channel along lines drawn through optical sections. Multichannel images were separated into single channels and exported to the ImageJ software (National Institute of Health, Bethesda, MD, USA). Measurements of the pixel intensities were made along the lines shown in the respective picture.

### Enzyme-Linked Immunosorbent Assays (ELISA)

MEFs were plated in 24-well plates at a cell density of 2.10^5 ^cells per well. Eight hours later, cells were infected with SeV or transfected with poly(I:C). Supernatants of cells were collected and ELISA assay was performed following the manufacter's protocol (PBL Biomedical Laboratories, Piscataway Township, NJ, USA, Mouse Interferon Beta ELISA Kit v.1.4 and R&D Systems, Mouse IL-6 immunoassay).

### Small interfering RNA (siRNA)

For down-regulation of proteins, siRNA oligos directed against MAVS, TRIM25, RIG-I and MDA-5 at a final concentration of 20 nM were transfected into cells for 72 hr. For HeLa cells, oligofectamine was used according to the manufacturer's instructions whereas for HEK293T cells, siRNA transfection was performed using the calcium phosphate precipitation method. siRNAs were purchased from Ambion (Life Technologies, Grand Island, NY, US). The sequence of the siRNA oligos is as follows (only sense strands are shown):

MAVS siRNA1: CCGUUUGCUGAAGACAAGAtt

MAVS siRNA2: CCACCUUGAUGCCUGUGAAtt

TRIM25 siRNAa: CCAUAGACCUCAAAAACGAtt

TRIM25 siRNAb: CAACAAGAAUACACGGAAAtt

RIG-I siRNA: GGAAGAGGUGCAGUAUAUUtt

MDA-5 siRNA: GUUCAGGAGUUAUCGAACAtt

### Mass spectrometry

After SeV H4 infection for four hours, a purified MAVS complex was analyzed by means of one-dimensional gel electrophoresis in combination with the nano liquid-chromatography tandem using 10 segment GelC/MS and spectral counting by mass spectrometry. Mass spectrometry was performed by Nextgensciences (Ann Arbor, MI, USA).

### Cellular fractionation

Isolation of mitochondrial and cytosolic fraction: HeLa cells were harvested in isotonic buffer-C (210 mM mannitol, 70 mM sucrose, 1 mM EDTA and 10 mM HEPES (pH 7.5)), supplemented with the protease inhibitor mixture Complete (Roche Molecular Biochemicals). Cells were broken by 15 passages through a 25-gauge needle fitted onto a 5 ml syringe, and the suspension was then centrifuged at 2,000 *g *at 4°C for 5 minutes to remove nuclei and unbroken cells. This procedure was repeated until nearly all of the cells were broken. Heavy membrane fractions enriched in mitochondria were obtained by centrifugation at 10,000 g at 4°C for 10 minutes, and supernatant was centrifuged at 25,000 *g *for 30 minutes and supernatant was kept as the "cytosolic fraction". The heavy membrane fraction was resuspended in buffer-C and layered on top of a discontinuous sucrose gradient consisting of 1.2 M sucrose in 10 mM Hepes (pH 7.5), 1 mM EDTA, and 0.1% BSA on top of 1.6 M sucrose in 10 mM Hepes, (pH 7.5), 1 mM EDTA, and 0.1% BSA. Then, samples are centrifuged at 30,000 *g *for 2 hr at 4°C. Mitochondria are recovered at the 1.6 to 1.2 M sucrose interface, washed in buffer C and centrifuged at 13,000 g at 4°C for 10 minutes, and resuspended in buffer C. The mitochondrial pellet was lysed and used for immunoblot analyses.

### Densitometric image analysis

To measure the relative expression level of proteins in cell extracts, acquired images were densitometrically analyzed by using the ImageJ software.

### Statistical analyses

Data were compared using Student's *t*-test. Differences were considered to be significant if *P *< 0.05. ***P < 0.001, **0.001 <*P *< 0.01, *0.01 <*P *< 0.05. NS, not significant.

## Abbreviations

CARDs: Caspase activation and recruitment domains; IFN: interferon; IRF3: interferon regulatory factor (IRF)-3; ISREs: IFN-stimulated response elements; MDA-5: melanoma differentiation-associated gene-5; MEF: murine embryonic fibroblast; MOI: multiplicity of infection; NF-κB: nuclear factor-kappaB; PBS: phosphate buffered saline; PRRs: pattern-recognition receptors; RLRs: RIG-I-like receptors; RLU: relative fluorescence units; SeV: Sendaï virus; TLRs: Toll-like receptors; VSV: Vesicular Stomatitis Virus; WT: wild type.

## Conflict of interest

The authors declare that they have no competing interests.

## Authors' contributions

CC and DA conceived the project and designed experiments. CC, NZ, AP and DA performed experiments. CC, NB, AV and DA analyzed data. CC and DA wrote the manuscript. AV and DA supervised the project, while DG provided key reagents. All authors read and approved the final manuscript.

## Supplementary Material

Additional file 1 **Figure S1**. Analysis of MAVS by immunoblotting in the absence or the presence of SDS. HEK293T or HeLa cells were infected or not with SeV H4 for 10 hrs. Next, cells were lysed in lysis buffer supplemented or not with 3% SDS. MAVS was analyzed in cell extract by immunoblotting. Actin was used as a protein loading control. Click here for file

Additional file 2 **Figure S2**. Degradation of the larger MAVS isoform is independent of type I IFNs. **(A) **HEK293T cells were treated with IFN α2 or β for 8 and 16 hr. After treatment, RIG-I, MAVS, p-Stat1 and Stat1 were analyzed in cell extracts by immunoblotting. Tubulin was used as a protein loading control. **(B) **HEK293T cells were infected with SeV H4 or treated with IFN α2 for 8 hr. RIG-I, MAVS, p-Stat1, Stat1, p-IRF3 and IRF3 were analyzed in cell extracts by immunoblotting. Tubulin was used as a protein loading control. **(C) **HEK293T cells were infected or not with SeV H4 for 10 hr in the presence or the absence of a neutralizing antibody raised against IFNAR1 (50 μg/ml). Next MAVS, p-Stat1 and Stat1 were analyzed in cell extracts by immunoblotting. Tubulin was used as a protein loading control. Click here for file

Additional file 3 **Figure S3**. Degradation of the larger MAVS isoform is independent of specific proteases. **(A) **HeLa cells were infected with SeV H4 or treated by staurosporine in the presence or the absence of the caspase inhibitor zVAD-fmk. Next, at various times, MAVS and PARP were analyzed by immunoblotting. Actin was used as a protein loading control. **(B) **HEK293T cells were transfected either with an IFN-β promoter reporter or with NF-κB reporter as well as with renilla luciferase as an internal control. Twenty hours after transfection, cells were infected with SeV WT or SeV H4 or else left non-infected and treated with caspase inhibitors. Luciferase assays were performed 8 hr after infection and was normalized using renilla luciferase activity. The error bars represent standard deviation from the mean value obtained from triplicate experiments. **(C) **HEK293T cells were infected with SeV H4 and treated with proteases inhibitors, and at various times after infection RIG-I, MAVS, p-IRF3, IRF3, p-IκBα and IκBα were analyzed in cell extracts by immunoblotting. Actin was used as a protein loading control. **(D) **HEK293T cells were transfected either with an IFN-β promoter reporter or with NF-κB reporter as well as with renilla luciferase as an internal control. Twenty hours after transfection, cells were infected with SeV WT or SeV H4 or else left non-infected and treated with leupeptin and pepstatin. Luciferase assays were performed 8 hr after infection and were normalized using renilla luciferase activity. Data represent means ± SD (*n *= 3). Click here for file

Additional file 4 **Figure S4**. MAVS is phosphorylated after RLR activation. **(A) **HeLa cells were infected with SeV H4. At various times after infection, MAVS was analyzed by immunoblotting. **(B) **HeLa cells were infected with SeV H4 for six hours. Next, endogenous MAVS was immunoprecipitated from cell extracts with a specific antibody. Following immunoprecipitation, samples were treated or not with λ phosphatase for 30 minutes. The presence of MAVS and its phosphorylation was examined by immunoblotting. Arrows indicate the phosphorylation of MAVS. Click here for file
