# Supplementary figures and images for: MAVS ubiquitination by the E3 ligase TRIM25 and degradation by the proteasome is involved in type I interferon production after activation of the antiviral RIG-I-like receptors
Source: BMC Biol. 2012 May 24;10:44. doi: 10.1186/1741-7007-10-44 (PMC3372421; doi:10.1186/1741-7007-10-44)

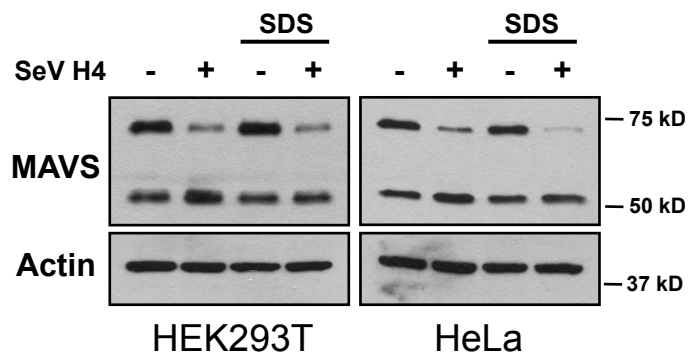

**Figure S1**

Supplement: Additional file 1 — Figure S1. Analysis of MAVS by immunoblotting in the absence or the presence of SDS. HEK293T or HeLa cells were infected or not with SeV H4 for 10 hrs. Next, cells were lysed in lysis buffer supplemented or not with 3% SDS. MAVS was analyzed in cell extract by immunoblotting. Actin was used as a protein loading control. [file 1741-7007-10-44-S1.PDF]

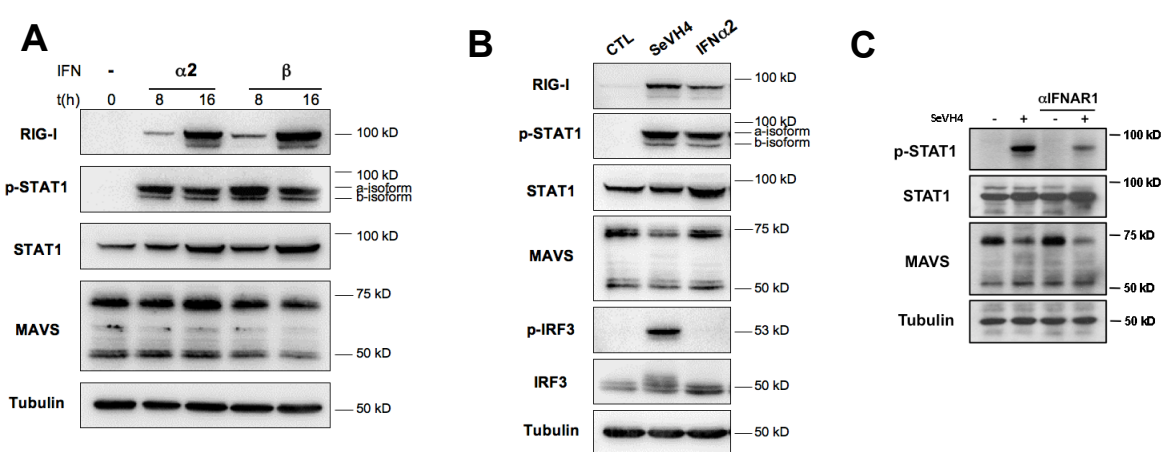

**Figure S2**

Supplement: Additional file 2 — Figure S2. Degradation of the larger MAVS isoform is independent of type I IFNs. (A) HEK293T cells were treated with IFN α2 or β for 8 and 16 hr. After treatment, RIG-I, MAVS, p-Stat1 and Stat1 were analyzed in cell extracts by immunoblotting. Tubulin was used as a protein loading control. (B) HEK293T cells were infected with SeV H4 or treated with IFN α2 for 8 hr. RIG-I, MAVS, p-Stat1, Stat1, p-IRF3 and IRF3 were analyzed in cell extracts by immunoblotting. Tubulin was used as a protein loading control. (C) HEK293T cells were infected or not with SeV H4 for 10 hr in the presence or the absence of a neutralizing antibody raised against IFNAR1 (50 μg/ml). Next MAVS, p-Stat1 and Stat1 were analyzed in cell extracts by immunoblotting. Tubulin was used as a protein loading control. [file 1741-7007-10-44-S2.PDF]

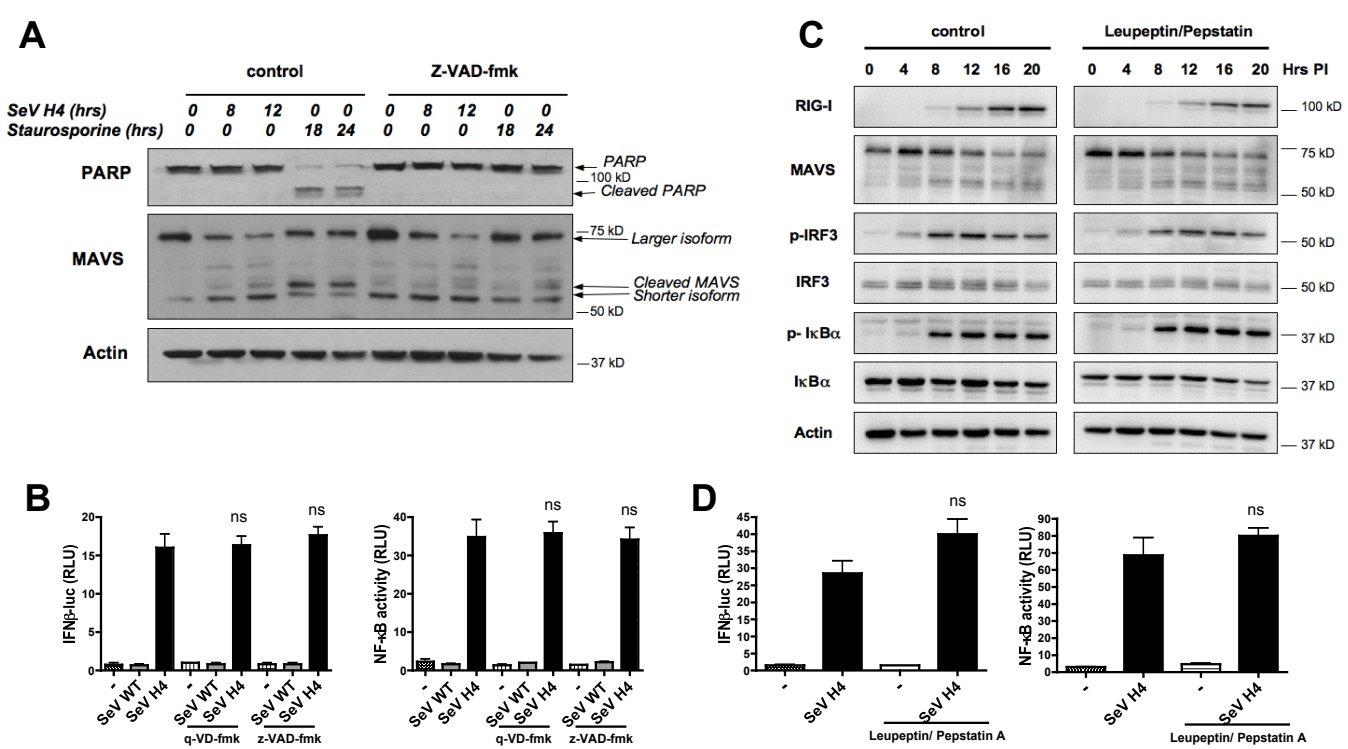

**Figure S3**

Supplement: Additional file 3 — Figure S3. Degradation of the larger MAVS isoform is independent of specific proteases. (A) HeLa cells were infected with SeV H4 or treated by staurosporine in the presence or the absence of the caspase inhibitor zVAD-fmk. Next, at various times, MAVS and PARP were analyzed by immunoblotting. Actin was used as a protein loading control. (B) HEK293T cells were transfected either with an IFN-β promoter reporter or with NF-κB reporter as well as with renilla luciferase as an internal control. Twenty hours after transfection, cells were infected with SeV WT or SeV H4 or else left non-infected and treated with caspase inhibitors. Luciferase assays were performed 8 hr after infection and was normalized using renilla luciferase activity. The error bars represent standard deviation from the mean value obtained from triplicate experiments. (C) HEK293T cells were infected with SeV H4 and treated with proteases inhibitors, and at various times after infection RIG-I, MAVS, p-IRF3, IRF3, p-IκBα and IκBα were analyzed in cell extracts by immunoblotting. Actin was used as a protein loading control. (D) HEK293T cells were transfected either with an IFN-β promoter reporter or with NF-κB reporter as well as with renilla luciferase as an internal control. Twenty hours after transfection, cells were infected with SeV WT or SeV H4 or else left non-infected and treated with leupeptin and pepstatin. Luciferase assays were performed 8 hr after infection and were normalized using renilla luciferase activity. Data represent means ± SD (n = 3). [file 1741-7007-10-44-S3.PDF]

**A**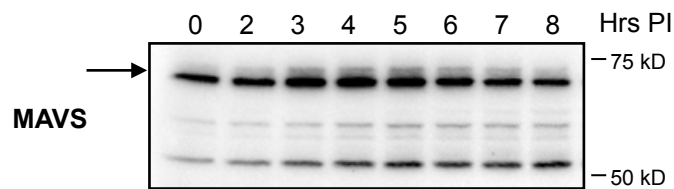**B**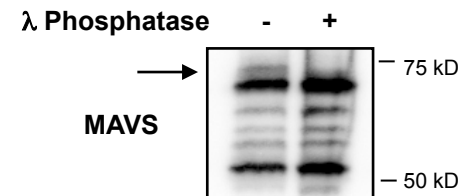**Figure S4**

Supplement: Additional file 4 — Figure S4. MAVS is phosphorylated after RLR activation. (A) HeLa cells were infected with SeV H4. At various times after infection, MAVS was analyzed by immunoblotting. (B) HeLa cells were infected with SeV H4 for six hours. Next, endogenous MAVS was immunoprecipitated from cell extracts with a specific antibody. Following immunoprecipitation, samples were treated or not with λ phosphatase for 30 minutes. The presence of MAVS and its phosphorylation was examined by immunoblotting. Arrows indicate the phosphorylation of MAVS. [file 1741-7007-10-44-S4.PDF]
